# Increased expression of adenosine 2A receptors in metastatic renal cell carcinoma is associated with poorer response to anti-vascular endothelial growth factor agents and anti-PD-1/Anti-CTLA4 antibodies and shorter survival

**DOI:** 10.1007/s00262-020-02843-x

**Published:** 2021-01-08

**Authors:** Takao Kamai, Toshiki Kijima, Toyonori Tsuzuki, Akinori Nukui, Hideyuki Abe, Kyoko Arai, Ken-Ichiro Yoshida

**Affiliations:** 1 Department of Urology, Dokkyo Medical University, 880 Kitakobayashi Mibu, Tochigi, 321-0293 Japan; 2 Department of Surgical Pathology, Aichi Medical University, Nagakute, Aichi Japan

**Keywords:** Adenosine 2A receptor (A2AR), CD39, CD73, PD-L1, Renal cell carcinoma

## Abstract

**Background:**

Adenosine and its adenosine 2A receptors (A2AR) mediate the immunosuppressive mechanism by which tumors escape immunosurveillance and impede anti-tumor immunity within the tumor microenvironment. However, we do not know whether the adenosine pathway (CD39/CD73/A2AR) plays a role in renal cell carcinoma (RCC). Therefore, we studied the role of immunosuppression in RCC by assessing the adenosine pathway in patients with RCC treated with anti-vascular endothelial growth factor (anti-VEGF) agents or immune checkpoints inhibitors (ICIs) or both.

**Methods:**

In 60 patients with metastatic RCC, we examined the expression of CD39, CD73, A2AR, and programmed cell death 1 ligand 1 (PD-L1) immunohistochemically in surgically resected tumor tissues and studied the clinicopathological characteristics of these patients. Patients were treated by cytoreductive nephrectomy with systemic therapy with anti-VEGF agent or a combination of the ICIs anti-cytotoxic T-lymphocyte-associated antigen 4 (CTLA4) antibody and programmed cell death 1 (PD-1) antibody.

**Results:**

Increased expression of A2AR in the primary tumors was associated with metastatic profiles. Patients treated with anti–PD-1 antibody in monotherapy, a combination of anti-PD-1 and anti-CTLA4 antibodies, or anti-VEGF agents showed better response and longer overall survival if the primary tumor had higher PD-L1 expression and lower A2AR expression. In Cox multivariate regression analysis, higher expression of A2AR was associated with shorter overall survival.

**Conclusions:**

Our findings suggest that the expression of A2AR and PD-L1 in the primary tumors in RCC might predict the outcomes of treatment with anti-VEGF agents and ICIs and that the A2AR pathway might be a molecular target for immunotherapy.

**Supplementary Information:**

The online version contains supplementary material available at 10.1007/s00262-020-02843-x.

## Introduction

Renal cell carcinoma (RCC) is immunogenic and proangiogenic. The introduction of anti-vascular endothelial growth factor (VEGF) therapies as a new treatment for metastatic RCC has improved the prospects of patients with this disease. However, many tumors eventually develop resistance to such therapies due to secondary mutation of the target protein or compensatory changes [1]. Accordingly, interest has grown in immunotherapy as an alternative to conventional treatment with cytokines such as interferon and interleukin two. The immune system is theoretically capable of suppressing tumor development and mediating tumor regression, but this process requires that tumor antigen-specific T cells are generated and activated. Thus, tumor cells can survive if they avoid being destroyed by the immune system, an ability that is now considered as one of the hallmarks of cancer [2].

The immune modulation mediated by the immune checkpoint pathway ensures that tissue is protected from collateral damage during an inflammatory response. The B7 family of immune-regulatory ligands, such as cytotoxic T-lymphocyte-associated antigen 4 (CTLA4) and programmed cell death 1 (PD-1)/PD-ligand 1 (PD-L1), are key players in immune checkpoints that positively or negatively regulate various immune responses, and tumor cells use this regulatory mechanism to evade a tumor-directed T-cell response by upregulating CTLA4 or PD-1/PD-L1 [3, 4]. Targeting the inhibitory CTLA4 or PD-1/PD-L1 with monoclonal antibodies has shown striking antitumor activity in patients with cancers, and a number of these objective responses seemed to be durable. Thus, immune checkpoint blockade may be a new standard for the treatment of cancer and have prospects for long-term clinical benefit, including advanced RCC [5, 6]. However, cancer cells develop innate or adaptive immune resistance and progress while being treated with anti-VEGF therapy or immune checkpoints inhibitors (ICIs), such as an anti-PD-1 antibody or an anti-CTLA4 antibody [3, 4].

Multiple immunosuppressive mechanisms impede anti-tumor immunity. These mechanisms include the accumulation of extracellular adenosine through the activation of purinergic receptors, a potent and widespread strategy exploited by tumors to escape immunosurveillance [7, 8]. Adenosine is a purine nucleotide released by injured tissue that decreases inflammation and protects tissue from immune-mediated damage [9–11]. Intracellular adenosine is metabolized to adenosine monophosphate (AMP) and inosine by adenosine kinase and adenosine deaminase. Adenosine triphosphate (ATP) and adenosine diphosphate (ADP) released outside the cell are dephosphorylated by membrane-bound ectonucleoside triphosphate diphosphohydrolase 1 (CD39) and ecto-5′-nucleotidase (CD73) localized on the cell surface and are converted to extracellular adenosine [8–11]. In recent years, ATP released by cytotoxicity was shown to cause an inflammatory response via ATP receptors on the cell membrane [12, 13]. Among four cell membrane adenosine receptors (A1, A2A, A2B, A3A), A2A adenosine receptor (A2AR) has the high affinity for adenosine and is present on cells of both the innate and the adaptive immune systems [14], and engagement of A2AR is a critical regulatory mechanism that protects tissues against excessive immune reactions [9–11]. In tumors, this pathway is hijacked and hinders anti-tumor immunity, promoting cancer progression [9–11]. Studies in animal models have shown that prior treatment with anti–PD-1 antibodies results in increased expression of A2AR and CD73, suggesting that the adenosine pathway may contribute to therapeutic resistance to immunotherapy [15, 16] Accordingly, interest has been increasing in new immunotherapy modalities targeting the adenosine pathway (CD39/CD73/A2AR) for cancer treatment [9–11].

Regarding the adenosine pathway in RCC, studies have shown that high CD73 expression correlates with poor prognosis [17] and that the *ADORA2A* (A2AR) and *NT5E* (CD73) genes are both highly expressed in the disease [18]. Recently, a phase I clinical trial with a small-molecule A2AR antagonist showed that this molecule could safely block adenosine signaling in a cohort of 68 RCC patients who had progressed on PD-1/PD-L1 inhibitors [18]. Thus, the adenosine pathway may play roles in the tumor microenvironment in RCC, and may be attractive for an immunotherapy. Therefore, we studied the relationship between the expression levels of PD-L1, CD39, CD73, and A2AR and clinicopathological features in patients with metastatic RCC who undertook cytoreductive nephrectomy and treated by anti-VEGF therapy or with an anti–PD-1 antibody monotherapy or a combination of anti-CTLA4 and anti–PD-1 antibodies. Such information should shed light on the role and biological significance of the adenosine pathway in RCC.

## Materials and methods

### Patients

This study was performed with data and surgical samples from 60 patients (43 men and 17 women; median age, 65 years; range 17–79 years) with histopathologically diagnosed metastatic RCC treated by cytoreductive nephrectomy at our center from 2011 to 2019. The types of RCC were as follows: clear cell RCC (ccRCC), 50 patients; papillary RCC type 2 (pRCC2), four patients; collecting duct RCC (coRCC), two patients; papillary RCC type 1 (pRCC1), one patient; chromophobe RCC (chRCC), one patient; sarcomatoid RCC, one patient; and RCC with Xp11 translocation involving TFE3 gene fusion (TFE-3 RCC), one patient. We also resected ten metastatic lesions during cytoreductive nephrectomy in ten patients, including seven distant organs and three lymph nodes.

All 60 patients treated by cytoreductive nephrectomy underwent preoperative computed tomography or magnetic resonance imaging or both for staging of the primary tumor. After cytoreductive nephrectomy, 49 patients were treated sunitinib or pazopanib as first-line systemic VEGF-targeting therapy for metastases and 11 patients were treated with a combination of anti-CTLA4 and anti–PD-1 antibodies. Of the 49 patients treated with an anti-VEGF agent, 20 were subsequently treated with anti–PD-1 antibody monotherapy as a second- or third-line treatment.

We also examined primary renal tumor samples from five patients with metastatic RCC (two ccRCC, two pRCC2, and one coRCC) who did not undergo cytoreductive nephrectomy and were treated with a combination of anti-CTLA4 and anti-PD-1 antibodies as first-line therapy. The primary renal tumors in these five patients were assessed by core needle biopsies.

Treatment effects were assessed according to the Response Evaluation Criteria in Solid Tumors (RECIST) criteria. Follow-up ranged from 3 to 77 months, with a median of 29 months. To assess metastatic lesions, computed tomography or magnetic resonance imaging or both was performed every 2–4 months.

To obtain the data for this study, we reviewed all participants’ medical records in March 2020. The study was conducted in accordance with the Declaration of Helsinki and was approved by the ethical review board of Dokkyo Medical University Hospital. Each patient provided written informed consent by signing a consent form that was approved by our institutional Committee on Human Rights in Research.

### Immunohistochemistry

Antibodies against CD8 (PA0183, Leica Biosystems Newcastle Ltd), PD-L1 (OptiView PD-L1 [SP142]; Ventana Medical Systems, Inc.), CD39 and CD73 (D7F9A; Cell Signaling Technology), and A2AR (SA654; ENZO Life Science) were used in formalin-fixed, paraffin-embedded tissues for immunohistochemical staining with the automated BOND system (Leica BOND-IIII system, Leica Biosystems Newcastle Ltd), as described previously [19]. Three of the authors independently examined 1500–2000 cancer cells and tumor-infiltrating lymphocytes (infiltrating immune cells that have a small, rounded cell, and a large dark-stained nucleus with little eosinophilic cytoplasm) in 7–10 microscopic fields of 2–3 slide sections of the eosin-stained slides and the identical immunostaining slides in a high-power view. The investigators rated the level of expression of PD-L1, CD39, CD73, and A2AR in the tumor cells (TC) and infiltrating immune cells (IC) on a score of 0–3, as follows: < 1% staining, TC-0/IC-0; 1–5% staining, TC-1/IC-1; 5–30% staining, TC-2/IC-2; and > 30% staining, TC-3/IC-3. Subsequently, the tumors were divided into two groups: a low expression group in which TCs and ICs were negative or weakly positive for the antibodies (< 5% of both of TCs and ICs were positive, TC-1 or IC-1; TC-0 or IC-0), and a high expression group in which TCs or ICs showed strong positivity for the antibodies (> 30% of either TCs or ICs, or both were positive, TC-3 or IC-3; TC-2 or IC-2), as performed in our previous study [20]. Accordingly, we evaluated the response rate and overall survival in the high expression and low expression groups.

We also examined the expression of PD-L1, CD39, CD73, and A2AR in 10 resected metastatic tumors from ten patients.

### Statistical analysis

We used Fisher’s exact test to determine whether the two categorical variables were associated with each other. The Kaplan–Meier method was used to create overall survival curves, and differences between the curves were assessed with the log-rank test. We examined prognostic factors affecting survival by Cox regression analysis. In all analyses, *P* < 0.05 indicated statistical significance. Analyses were performed with commercial software.

## Results

**Fig. 1:**
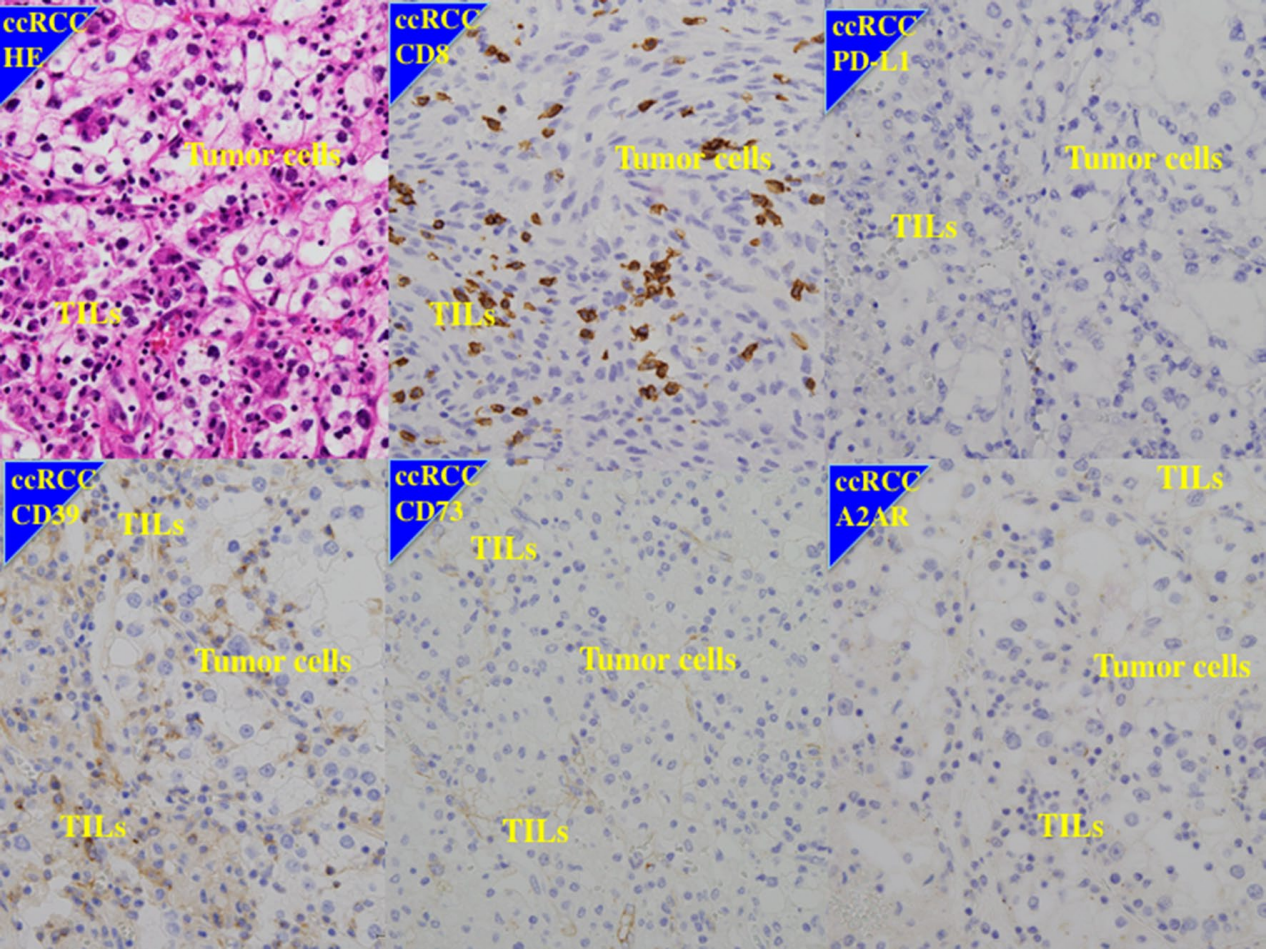
Immunohistochemistry of a case of better response to anti-VEGF therapies and longer survival. 73 y.o. male of left clear cell renal cell carcinoma with Fuhrman grade 2, pT2bN0M1 (PUL, LYM). CD8; high expression, PD-L1; low expression, CD39: high expression, CD73; low expression, A2AR; low expression. After cytoreductive nephrectomy, this patient had received sunitinib as first-line anti-VEGF therapy for 17 months with best response of partial response. After refractory to sunitinib, he has received axitinib as second-line anti-VEGF therapy with alive with disease for 29 months

**Fig. 2:**
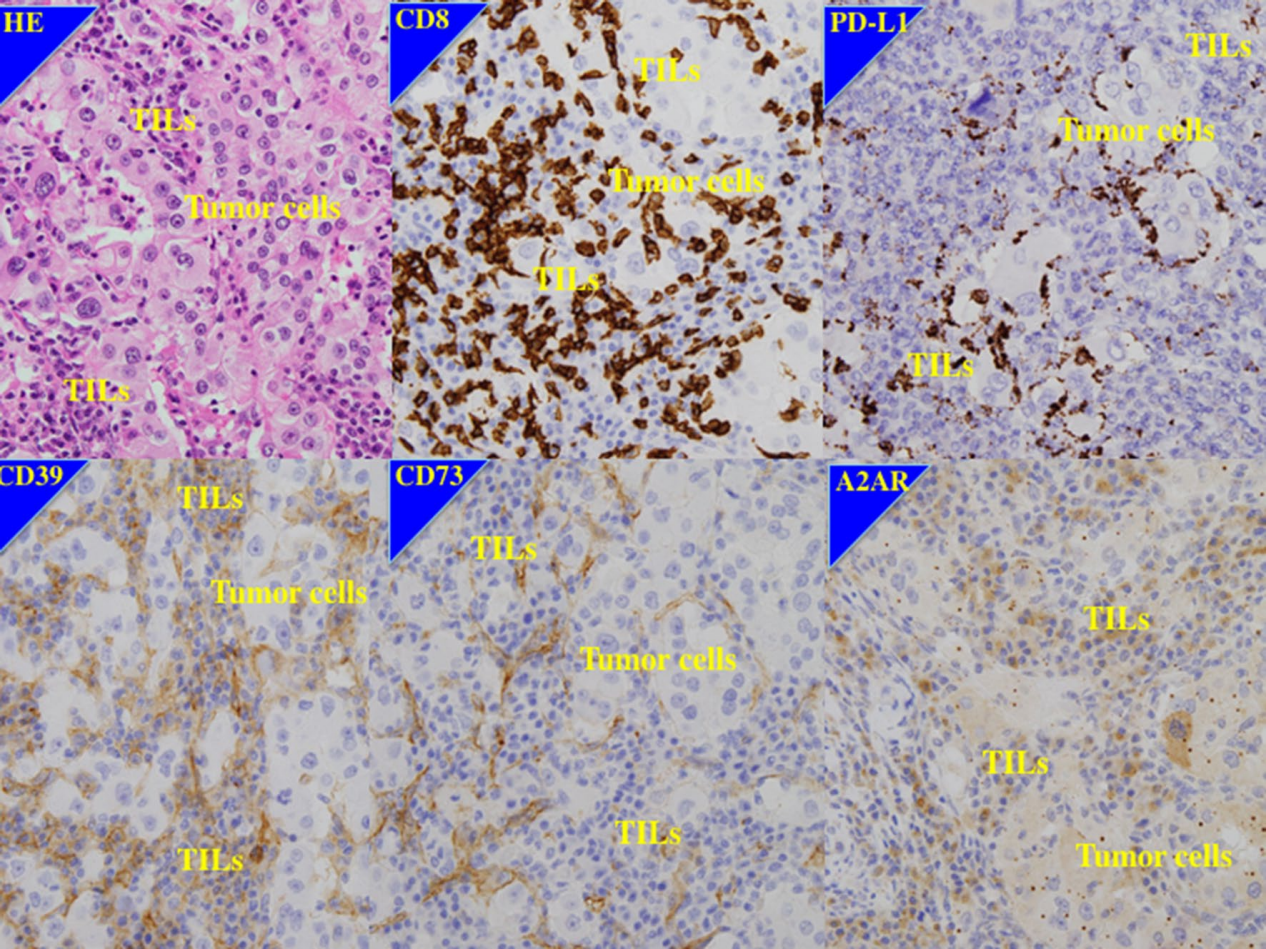
Immunohistochemistry of a case of worse response to first-line anti-VEGF therapy and second-line nivolumab and shorter survival. 64 year old male of right clear cell renal cell carcinoma with Fuhrman grade 3, pT2aN0M1 (PUL, HEP). CD8; high expression, PD-L1; high expression, CD39: high expression, CD73; high expression, A2AR; high expression. After cytoreductive nephrectomy, this patient had received pazopanib as first-line anti-VEGF therapy for 3 months, while the metastatic disease progressed. Then the patient received nivolumab as second-line therapy for 3 months. The best response was partial response for pulmonary and liver lesions, however, the lesions progressed rapidly and the patient dead

**Fig. 3:**
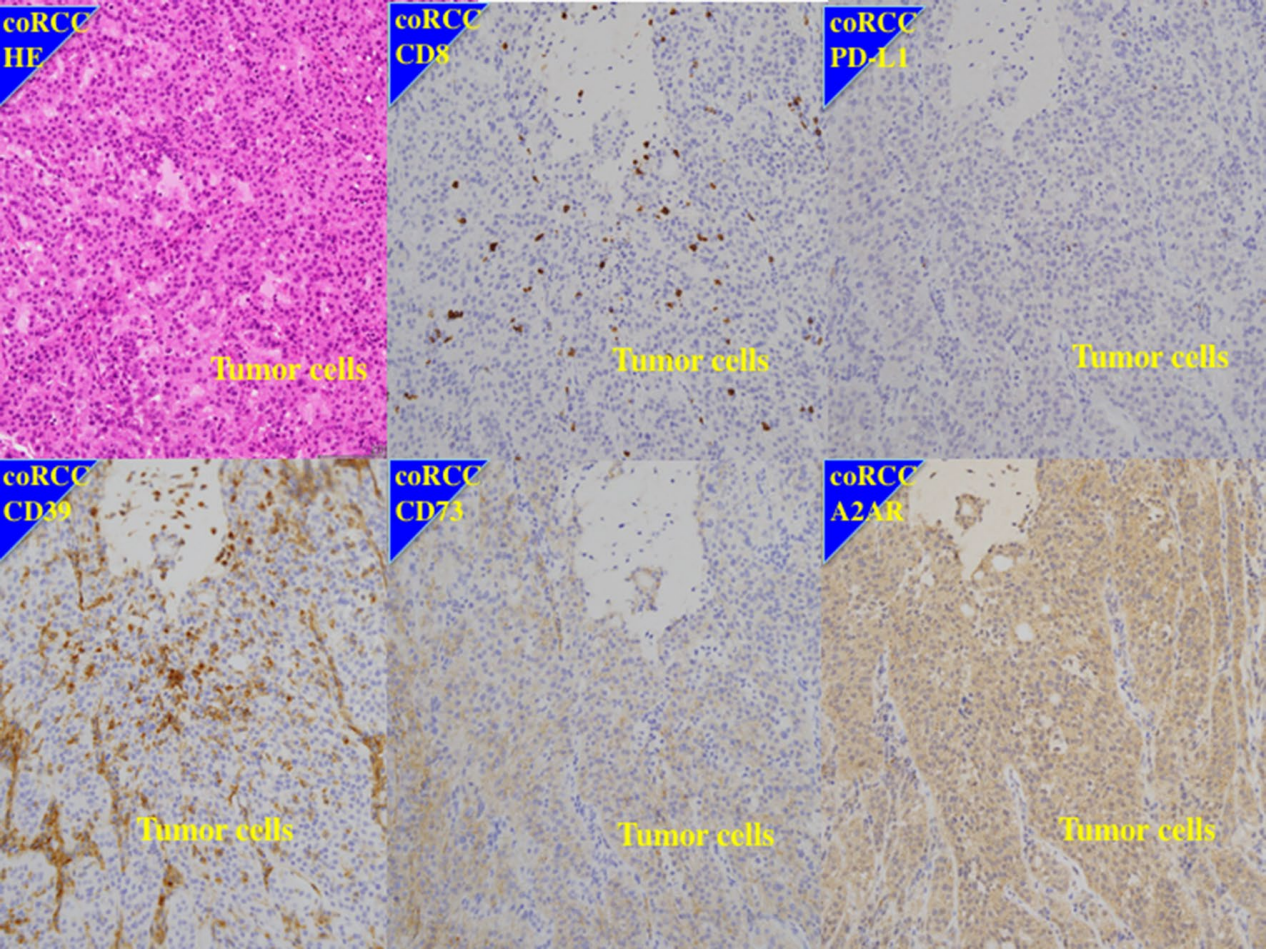
Immunohistochemistry of a case of worse response to first-line combination of ipilimumab and nivolumab and shorter survival. 57 year old male of right collecting duct renal cell carcinoma with Fuhrman grade 3, pT2aN0M1 (PUL, HEP). CD8; low expression, PD-L1; low expression, CD39: high expression, CD73; high expression, A2AR; high expression. After cytoreductive nephrectomy, the patient had received combination of ipilimumab and nivolumab as first-line therapy. He showed no response to the therapy and dead after 4 months

**Fig. 4:**
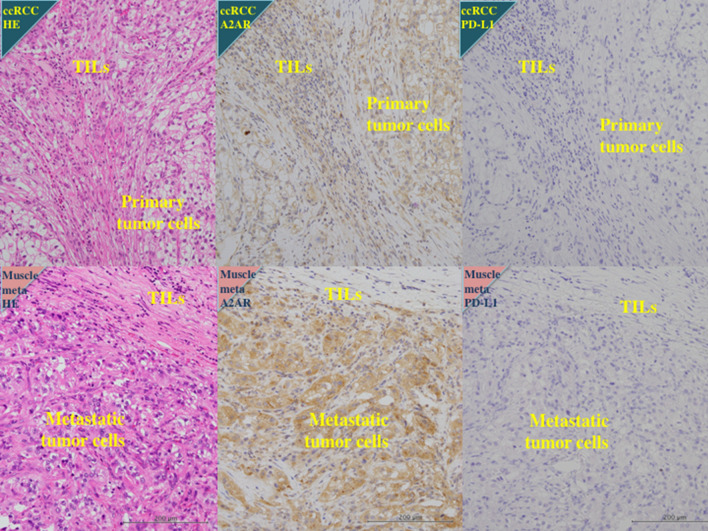
Immunohistochemistry of clear cell RCC with muscle and pulmonary metastases. 71 y.o. male of left clear cell renal cell carcinoma with Fuhrman grade 3, pT1aN0M1 (PUL, OTH). Primary renal tumor showed high expression for A2AR and negative staining for PD-L1. Metastatic tumor in psoas major muscle showed high reaction for A2AR and negative reaction for PD-L1. After cytoreductive nephrectomy and resection of metastatic tumor in psoas major muscle, this patient had received sunitinib as first-line anti-VEGF therapy for 5 months, but pulmonary disease progressed slowly. Then the patient has received nivolumab as second-line therapy. He showed no response to the therapy and dead after 21 months

Many of the cells that showed a positive reaction for anti–PD-L1, anti-CD8, anti-CD39, anti-CD73, and anti-A2AR antibodies were immune cells (Figs. 1, 2, 3, Supplementary Figs. 1, 2), although some tumor cells also showed a positive reaction for these antibodies, in particular for anti-A2AR antibody (Figs. 2, 3, 4, Supplementary Figs. 1, 2).

Increased expression of A2AR in the primary tumors was significantly associated with metastatic profiles for several organs (*P* = 0.0002) but was not significantly associated with regional lymph node involvement (*P* = 0.0744, Table 1). There was no relationship between expression of A2AR and histological grade or local invasion (Table 1). Compared with ccRCC, non-clear cell RCC (non-ccRCC) had a higher expression of A2AR (*P* = 0.0071) and lower expression of PD-L1 (*P* = 0.0109); however, we found no difference between RCC histological types and expressions for CD39 (*P* = 0.4058) or CD73 (*P* = 0.2963) (Fig. 3, Supplementary Figs. 3–5).

**Table 1:** Relationship between PD-L1/CD39/CD73/A2AR status and clinicopathological characteristics

		Grade		pT		pN		Metastatic organs*			anti-VEGF		ICI		anti-VEGF + / − ICI	
		1,2	3,4	1,2	3.4	0	1,2	Pul	Pul/Oth	Oth	PR/SD	PD	PR/SD	PD	PR/SD	PD
		(*n* = 12)	(*n* = 48)	(*n* = 6)	(*n* = 54)	(*n* = 39)	(*n* = 21)	(*n* = 14)	(*n* = 28)	(*n* = 18)	(*n* = 26)	(*n* = 23)	(*n* = 13)	(*n* = 18)	(*n* = 34)	(*n* = 26)
Grade	1,2 (*n* = 12)							6	3	3	8	2	2	0	10	2
	3,4 (*n* = 48)							8	25	15	18	21	11	18	24	24
	*p* value							0.0449			0.0210		0.0635		0.0518	
pT	1,2 (*n* = 6)							2	3	1	6	1	3	0	5	1
	3,4 (*n* = 54)							12	25	17	20	22	10	18	29	25
	*p* value							0.7059			0.3532		0.0637		0.2206	
pN	0 (*n* = 39)							13	12	14	22	9	12	9	27	12
	1,2 (*n* = 21)							1	16	4	4	14	1	9	7	14
	*p* value							0.0024			0.0013		0.0200		0.0132	
Meta organs	Pul (*n* = 14)										14	0	5	0	14	0
	Pul/Oth (*n* = 28)										10	10	6	9	15	13
	Oth (*n* = 18)										2	13	2	11	5	13
	*p* value										< 0.0001		0.0306		0.0002	
PD-L1	low (*n* = 32)	7	25	2	30	20	14	5	13	14	12	16	4	14	15	17
	high (*n* = 28)	5	23	4	24	19	9	9	15	4	14	7	9	4	19	9
	*p* value	0.7560		0.4039		0.7879		0.0768			0.1488		0.0130		0.1232	
CD39	low (*n* = 23)	6	17	2	21	15	8	6	12	5	11	5	6	5	15	8
	high (*n* = 37)	6	31	4	33	24	13	8	16	13	15	18	7	13	19	18
	*p* value	0.5081		0.7906		0.9778		0.5455			0.1431		0.4491		0.4221	
CD73	low (*n* = 44)	9	35	5	39	31	13	12	19	13	20	17	10	12	28	14
	high (n = 16)	3	13	1	15	8	8	2	9	5	6	6	3	6	6	12
	*p* value	0.8839		0.5593		0.2203		0.4634			0.8068		0.5347		0.2234	
A2AR	low (*n* = 35)	9	26	4	31	26	9	13	18	4	23	8	10	3	26	9
	high (*n* = 25)	3	22	2	23	13	12	1	10	14	3	15	3	15	8	17
	*p* value	0.9208		0.6625		0.0744		0.0002			< 0.0001		0.0002		0.0002	
PD-L1 + A2AR	low-low (*n* = 13)	3	10	0	13	9	4	4	5	4	9	4	2	2	9	4
	low–high (*n* = 18)	4	14	2	16	10	8	1	7	10	0	9	3	12	6	12
	high-low (*n* = 22)	4	18	4	18	17	5	9	13	0	17	6	7	0	18	4
	high-high (*n* = 7)	1	6	0	7	3	4	0	3	4	0	4	1	4	1	6
	*p* value	0.9561		0.2743		0.3075		0.0021			0.0012		0.0009		0.0014	

*Pul* lung metastasis only, Pul/Oth; metastases to lung and othe organs, *Oth* other than lung metastases

Metastatic organs*

**Fig. 5:**
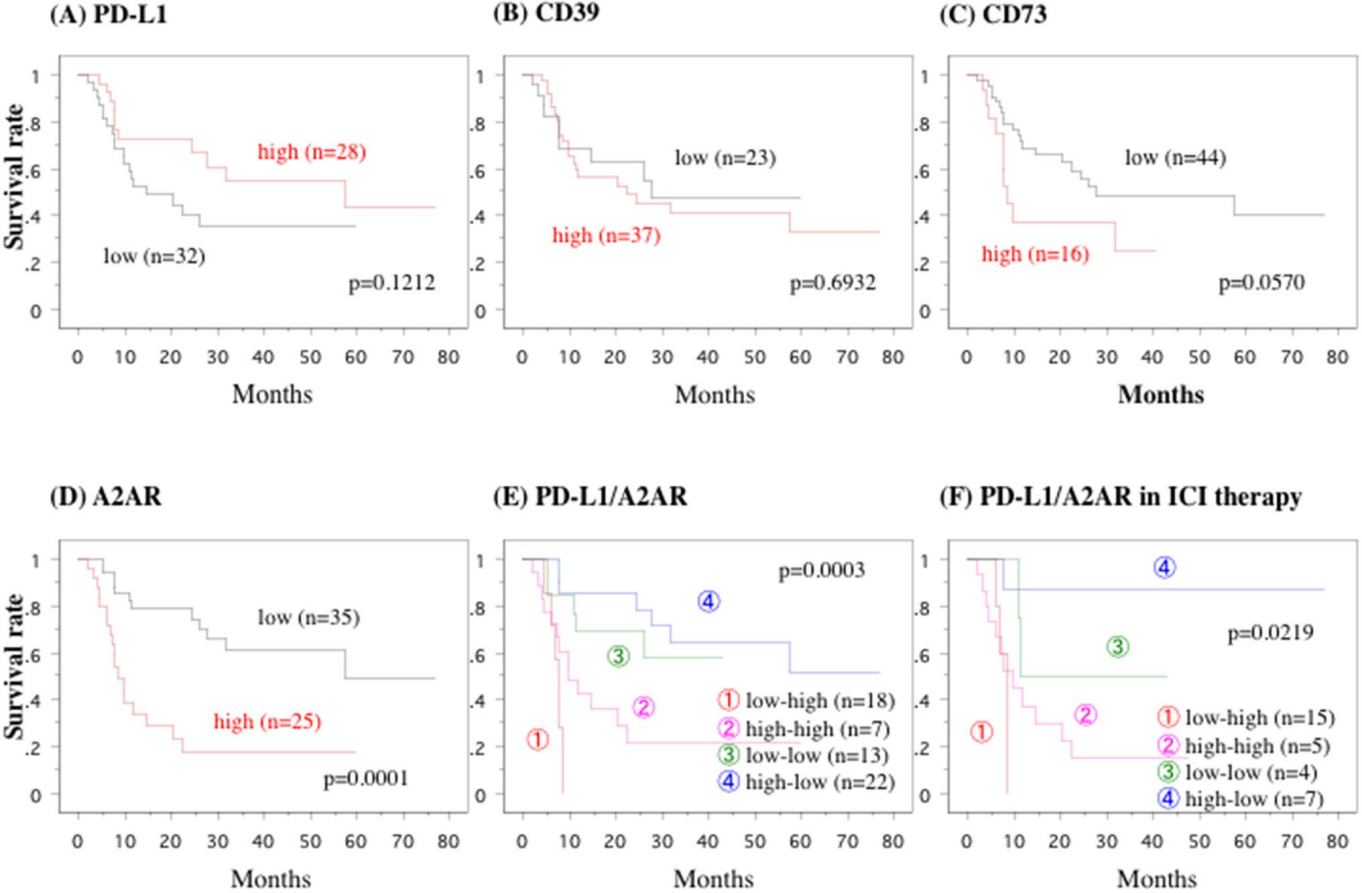
Overall survival curve. This overall survival curve is based on the expression status of PD-L1 (**a**), CD39 (**b**), CD73 (**c**), A2AR (**d**), combination of PD-L1 and A2AR in all patients (**e**) and in the patients treated with immune checkpoints inhibitors (ICIs) (**f**). In Figure E, in comparison to the patients in type I (PD-L1 low and A2AR high), the relative risk (RR) of the patients in type IV (PD-L1 high and A2AR low) was 0.107 (95% confidential intervals (CI); 0.030–0.380, *P* = 0.0005), RR in type III (PD-L1 low and A2AR low) was 0.134 (95%CI; 0.035–0.515, *P* = 0.0034), and RR in type II (PD-L1 high and A2AR high) was 0.468 (95%CI; 0.153–1.427, *P* = 0.1820). In Figure F, compared to the patients in low–high type-I, the relative risk (RR) of the patients in high-low type-IV was 0.060 (95% confidential intervals (CI); 0.006–0.628, *P* = 0.0108), RR in low-low type-III was 0.143 (95%CI; 0.014–1.512, *P* = 0.1060), and RR in high-high type-II was 0.595 (95%CI; 0.151–2.351, *p* = 0.4589)

In all 60 patients, neither histological grade nor pT stage influenced the effects of the treatments; however, patients with regional lymph node involvement had a poorer response in distant metastatic organs (*P* = 0.0132, Table 1). Regardless of whether patients were treated only with an anti-VEGF (*n* = 29 patients) agent, with an anti-VEGF agent followed by anti–PD-1 antibody monotherapy (*n* = 20), or only with anti–PD-1 and anti-CTLA4 antibodies (*n* = 11), patients with lower A2AR expression in the primary tumors had better response (complete response, partial response, or stable disease) to anti-VEGF agents (*P* < 0.0001) and ICIs (*P* = 0.0002) (Table 1) and longer survival (*P* = 0.0001) (Fig. 5).

Expressions of PD-L1, CD39, and CD73 were not related to histological grade, local invasion, regional lymph node involvement, or metastatic profiles (Table 1). The expression levels of PD-L1 in the primary tumors did not relate to the response to anti-VEGF therapies for metastatic lesions or survival in any of the 60 patients (Table 1 and Fig. 5); however, among the 31 patients who received ICIs, 13 patients with higher PD-L1 expression showed better response to ICIs (*P* = 0.0130) (Table 1) and a weak tendency towards longer survival (*P* = 0.1581). The primary tumors of 37 of the 60 patients showed higher expressions of CD39, but expression levels of CD39 had no relation to the response to the therapies or survival (Table 1 and Fig. 5). In contrast, the primary tumors in 44 patients had lower expression of CD73, which was weakly related to longer survival (*P* = 0.0570, Fig. 5), but not treatment response (Table 1). Although CD8-positive T cells were seen in many tumor microenvironments, expression of CD8 had no influence on histological grade, local invasion, regional lymph node involvement, or response to anti-VEGF agents or ICIs. On the other hand, our findings indicated that pulmonary metastatic lesions might be more closely linked to lower A2AR expression in primary renal tumor (*P* = 0.0002) and better response to anti-VEGF agents (*P* < 0.0001) and ICIs (*P* = 0.0002) than other metastatic lesions are (Table 1).

Our analysis of the expression of PD-L1, CD39, CD73, and A2AR in the 10 resected metastatic lesions showed a positive relation of the expression levels in the primary tumor and metastatic lesion for A2AR (*P* = 0.0157) but not for PD-L1 (*P* = 0.3894), CD39 (*P* = 0.5271), or CD73 (*P* = 0.1967). We found that 7 of these patients had a higher expression of A2AR in both the metastatic lesion and the primary renal tumor. In six of these seven patients, other nonresectable metastatic lesions showed poorer response to anti-VEGF agents and subsequent anti–PD-1 antibody monotherapy or a combination of anti–PD-1 and anti-CTLA4 antibodies, regardless of the expression of PD-L1 in the primary tumor and metastatic lesion (Fig. 4, Supplementary Fig. 6, 7); in contrast, the 3 patients with a lower expression of A2AR in the metastatic lesions and/or primary tumor had a higher PD-L1 expression in the metastatic lesions and showed better treatment response (Supplementary Fig. 8).

In the five patients with metastatic RCC treated with a combination of anti-CTLA4 and anti–PD-1 antibodies, we found no relationship between CD8-positive T cells in the tumor microenvironment in core-needle biopsy for primary renal tumors and treatment response. Two of the patients had lower A2AR expression, and their renal tumors and metastatic lesions showed partial remission or stable disease after treatment (Supplementary Fig. 9). The remaining three patients had higher A2AR expression, and two of these three patients showed stable response in some lesions or poorer response in others (Supplementary Fig. 9).

To study the roles of PD-L1 and A2AR, we combined the expression levels of PD-L1 and A2AR in the primary tumor and subsequently classified the patients into four groups: type I, PD-L1 low and A2AR high; type II, PD-L1 high and A2AR high; type III, PD-L1 low and A2AR low; and type IV, PD-L1 high and A2AR low. Type IV expression was associated with longer survival (Fig. 5e), in particular in patients who received ICIs (Fig. 5f). In contrast, patients with higher A2AR expression in the primary tumor showed worse response (progressive disease) to the anti-VEGF agents or ICIs (*P* = 0.0002, Table 1), and poorer overall survival (*P* = 0.0001, Fig. 5), regardless of expression of PD-L1.

When grade, pT stage, lymph node metastasis stage, PD-L1, CD39, CD73, A2AR and histology (ccRCC vs. non-ccRCC) were correlated in Cox univariate analysis, higher histological grade, higher pT stage, positive lymph node metastasis, and higher CD73 and A2AR were associated with shorter overall survival. In Cox multivariate analysis, A2AR and grade were significant (*P* = 0.0006, *P* = 0.0062, respectively, Table 2).

**Table 2:** Cox regression analysis for various potential prognostic factors in overall survival

Variable	Unfavorable/favorable characteristics	No. of patients	Univariate (U)			Multivariate (M)		
			Relative risk	95% confidential interval	*P* value	Relative risk	95% confidential interval	*P* value
Grade	4,3/2,1	48/12	6.33	1.45–26.82	0.0122	11.05	1.98–61.84	0.0062
pT	4,3/2,1	54/6	2.49	0.59–10.56	0.2131	1.05	0.21–5.25	0.9510
pN	2,1/0	21/39	3.00	1.45–6.21	0.0030	1.52	0.67–3.47	0.3208
PD-L1	low/high	32/28	0.56	0.26–1.18	0.1268	0.78	0.28–2.18	0.6422
CD39	high/low	37/23	1.16	0.54–2.49	0.6939	0.86	0.37–1.98	0.7164
CD73	high/low	16/44	2.08	0.96–4.50	0.0629	2.98	0.81–5.47	0.1297
A2AR	high/low	25/35	3.98	1.88–8.41	0.0003	6.89	2.28–20.89	0.0006
Histology	non-ccRCC/ccRCC	10/50	1.39	0.57–3.39	0.4742	1.01	0.34–2.91	0.9908

## Discussion

The present study examined the expression levels of PD-L1, CD39, CD73, and Although the expression pattern of these four molecules was diverse in individual cases, we found that higher A2AR expression in the primary tumor was associated with worse response to both anti-VEGF agents and ICIs. A higher A2AR expression in the primary tumor was also an independent prognostic factor for shorter survival. Furthermore, patients with higher PD-L1 expression in the primary tumor who were treated with ICIs showed a more favorable response but not a longer survival. Last, lower CD73 expression correlated with longer survival but not with treatment response. CD39 levels were not associated with treatment response or survival. These findings suggest that not only PD-L1 but also A2AR might be required in the tumor microenvironment to allow the tumor to evade the host immune system and that blockade of the A2AR pathway might be a new anti-tumor response for advanced RCC.

Because cancer cells evade the immune system by hijacking immune cells [2], elucidating the orchestrating mechanism between tumor and immune cells in the tumor microenvironment might be essential to enable cancer immunotherapy. The expression of PD-L1 on tumor cells has been associated with good prognosis and sustained clinical responses in immunotherapeutic regimens based on PD-L1/PD-1/CTLA4 immune checkpoint blockade; however, the relative importance of PD-L1 expression on tumor cells versus immune cells in the tumor microenvironment is a matter of controversy. Expression of PD-L1, CD39, CD73, and A2AR were increased in immune cells in the present study, while some tumor cells showed positive expression of A2AR. Although the different roles of the expression of PD-L1, CD39, CD73, and A2AR in tumor and immune cells were unclear, our findings suggested that the influence of the A2AR pathway and the PD-1/PD-L1 axis on the interaction between the immune system and tumor cells in the tumor microenvironment might be linked with cancer progression in RCC.

Patients who are resistant or refractory to anti–PD-1/PD-L1 antibodies or who have predominantly PD-L1–negative tumors might not harbor immune-suppressed characteristics through the PD-1/PD-L1 axis [3, 4]. In the present study, patients with higher expression of PD-L1 in the primary tumor showed a favorable response to ICIs regardless of the effects of previous anti-VEGF agents; however, they did not have a longer survival. In contrast, patients with higher expression of A2AR showed poorer response not only to ICIs but also to anti-VEGF agents and had a shorter survival, regardless of the expression levels of PD-L1. The biological characteristics of primary and metastatic tumors are known to not always be identical [21]. In the present study, metastatic lesions were resected in only ten cases. Nevertheless, we found that the primary renal tumors with a higher expression of A2AR had metastatic lesions with a higher expression of A2AR, but we did not find such a positive relation for PD-L1, CD39, or CD73. The nonresectable metastatic tumors in patients with a higher expression of A2AR in the resected metastatic tumor showed worse response, regardless of the expression level of PD-L1 in the resected metastatic tumor. In the present study, 21 patients with regional lymph node metastasis showed a worse treatment response for other distant nonresectable metastatic lesions. This might be linked with the following findings based on three resected metastatic lymph nodes from three patients. Two of these patients had higher A2AR expressions in the resected metastatic lymph nodes and the primary renal tumor. They showed a worse response of the nonresectable distant metastatic lesions to anti-VEGF agents or ICIs, regardless of the expression level of PD-L1. The third patient had a lower expression of A2AR in both the metastatic lymph node and the primary renal tumor, and showed better treatment response. These findings indicate that even if metastatic lesions in patients with a higher expression of PD-L1 in the primary tumors or metastatic tumors or both show better response to ICIs in the short term, the prognosis for survival might be worse than we expected. The findings also highlight the impact of A2AR on cancer progression in RCC. In fact, in a recent phase I clinical trial a small-molecule A2AR antagonist safely blocked adenosine signaling in a cohort of 68 patients with RCC refractory to PD-1/PD-L1 inhibitors [18]. Moreover, treatment with anti–PD-1 has been reported to lead to increased expression of A2AR and CD73 and to be associated with enhanced tumor responses to A2AR blockade [16]. Accordingly, cancer cells are likely to exploit the A2AR pathway to promote metastasis and progression, and an increased expression of A2AR not only in the primary tumor but also in metastatic lesions probably has an anti-immune effect that enables cancer cells to survive and develop resistance to anti-tumor agents. Because many of the primary tumors in our sample were not well histologically differentiated (48/60 tumors) and were locally invasive (54/60 tumors), future studies should study the expression of PD-L1, CD39, CD73, and A2AR in primary tumors with well-differentiated, non-invasive, and non-metastatic profiles and in distant metastatic lesions to understand the differences in tumor microenvironments between primary tumors and their metastatic lesions. A diverse expression pattern of PD-L1, CD39, CD73, and A2AR was found not only in each patient but also in inter-individual metastatic lesions. If possible, it may be useful to study the expression of these molecules in individual metastatic lesions and compare their expression to examine the immunological difference in the tumor microenvironments and to determine individual patient treatment strategies in the future.

Both the hypoxia-inducible factor (HIF)-VEGF pathway and PD-1/PD-L1 axis play essential roles in the progression of RCC. A study performed in an effort to develop a tolerable and more effective combination regimen of an anti-VEGF agent and an anti–PD-1 antibody found improved outcome [22, 23]. Our findings that increased expression of A2AR correlated with poorer response to anti-VEGF agents, as well as ICIs, indicates that in RCC A2AR might be associated with angiogenesis by an unknown mechanism. Several lines of studies have suggested that adenosine stimulate VEGF production by immune cells through the stimulation of A2AR [14]. Thus, it is likely that the immunosuppressive A2AR pathway influences angiogenesis. Furthermore, at present we do not know whether A2AR-related resistance to anti-VEGF agents and ICIs exists at the time of tumor diagnosis or evolves as a resistance mechanism during the course of treatment. The mechanisms by which blocking of the VEGF pathway and PD-1/PD-L1 axis may influence the biological profiles of PD-1, PD-L1, CD39, CD73, and A2AR in the tumor microenvironment are also unclear. Future research should study the molecules of the HIF-VEGF, PD-1/PD-L1, and A2AR pathways in RCCs to investigate the spatial and temporal biological changes in the primary and metastatic lesions before and after treatment.

In the present study, we found CD8-positive T cells in many tumor microenvironments, but expression of CD8 did not correlate with pathological status, response to anti-VEGF agents or ICIs, or survival. Antigen-specific CD8 T cells are known to play an important role in controlling cancer, but persistent antigen stimulation results in T-cell exhaustion, suggesting that some T cells are exhausted or inhibited after chronic antigen stimulation or metabolic reprogramming [24–28]. Exhausted CD8 T cells have decreased effector function and proliferative capacity, partly caused by overexpression of inhibitory receptors such as PD-1 [24–28]. Blockade of PD-1 reinvigorates T cell responses. Thus, elucidating roles of T-cell exhaustion in tumor microenvironments may be needed to understand the immune-suppressive mechanisms of tumors and develop immunotherapeutic interventions. In contrast, a recent phase I clinical trial with an A2AR antagonist found that long-term clinical benefit is associated with increased recruitment of CD8-positive T cells into the tumor [18].

As part of the present study, five patients with metastatic RCCs who did not undertake cytoreductive nephrectomy were treated with a combination of anti-CTLA4 and anti–PD-1 antibodies as the first-line therapy. In two patients with lower A2AR expression in the core-needle biopsies from the primary renal tumors, both the primary renal tumors and the metastatic lesions showed better response, whereas two of three patients with higher A2AR expression showed a poorer response. However, CD8-positive T cells in the tumor microenvironment were not related to treatment response. Thus, although the expression levels of CD8 had no association with histological grade, local invasion, regional lymph node involvement, or the effects of treatment, future studies should examine immune effector cytotoxic CD8-positive T cells and immune-suppression exhausted CD8 T cells before and after treatment with anti-VEGF agents and ICIs.

Adenosine signaling has been reported not only to directly dampen T-cell immunity but also to shift the balance away from T effector responses towards myeloid suppressor recruitment [9–11]. Furthermore, adenosine has been reported to drive naive CD4 T cells toward a regulatory phenotype, CD4-positive (CD4 + CD25 + Foxp3 +) regulatory T cells (Tregs), via induction of FoxP3, which directs macrophage differentiation towards an M2 phenotype, inhibits natural killer cell function and diminishes antigen presentation by dendritic cells [11, 29]. Tregs infiltrate tumors, and tumor cells that express CD39 and CD73 at high levels produce adenosine and suppress the tumor immune response through A2AR of effector T cells and natural killer cells. Adenosine produced by Tregs activates the A2AR on CD4 + CD25 effector T cells and suppresses the immune response [30]. On the other hand, anti-VEGF therapy can also decrease Treg, either by inhibiting accumulation of myeloid-derived suppressor cell (MDSC) and immature dendric cell (DC) in the tumor microenvironment or directly through VEGF pathway inhibition on Treg [31–33]. MDSCs are involved with the differentiation of antigen-presenting cells (APCs), such as macrophages and DCs. MDSCs, Tregs, DCs, and tumor-associated macrophages (TAMs) may enhance the tumor-promoting immune response and control cancer growth [34]. Therefore, further research is needed on the expression of A2AR in Treg, MDSCs, DCs, and TAMs to shed light on the tumor-promoting immune response of A2AR in these immune cells infiltrating tumor tissues and also to explore the interrelations between them in the tumor microenvironment.

RCCs have unique features that make them attractive for therapeutic approaches targeting components of the immune system. In the present study, patients with increased A2AR expression in the primary lesion showed worse response to anti-VEGF agents and ICIs and shorter survival. Although the spatial and temporal molecular biological differences between the primary and metastatic lesions are unknown, our findings might highlight the therapeutic potential of blocking adenosine-mediated immunosuppression to strengthen anti-tumor immunity. To elucidate how the CD39/CD73/A2AR pathway and PD-1/PD-L1 axis interact in immunosuppressive signaling in RCC, we need to study the mechanisms underlying anti-tumor immunity, including immune cell infiltration and angiogenesis. Without such information, we cannot effectively utilize PD-L1- or A2AR-mediated immunosuppression for cancer immunotherapy.

The limitations of the present study were its relatively small patient population, and short follow-up period, which did not allow us to draw definite conclusions from the results. As described above, another limitation was the lack of any mechanistic insight into how A2AR enhances anti-tumor immunity and the mechanism of suppressing T cell immunity through extracellular adenosine production and after binding to A2AR. T cells are activated by dendritic cells presenting non-self antigens, and the activated T cells migrate to target organs and damage tissues [3, 4]. Future studies should investigate the pharmacokinetics of adenosine, AMP, cyclic AMP, ADP, and ATP in tumor cells and immune cells to understand how the CD39/CD73/A2AR pathway in the tumor microenvironment plays a role in how cancer cells survive and escape the anti-tumor immune response. On the other hand, since tumor-related inflammation plays a vital role in the development and progression of cancers [35, 36], we should study the relationship between expression of CD39/CD73/A2AR and serum inflammatory markers, including the neutrophil-to-lymphocyte ratio (NLR), the platelet-to-lymphocyte ratio (PLR), the monocyte-to-lymphocyte ratio (MLR), C-reactive protein, and the systemic immune-inflammation index (SII), or a systemic inflammation response index (SIRI). We studied only 10 cases of non-ccRCC, so future studies should also examine the roles of CD39/CD73/A2AR in papillary, chromophobe, and collecting duct RCC in larger cohorts. In addition, in this study we evaluated the association only of adenosine with RCC, so future studies should also evaluate the roles of other nucleotides, such as guanine and pyrimidine nucleosides, in RCC. The results of such studies might be able to shed light on immunosuppressive signaling in human RCC and on how to select patients with tumors that are immune suppressed through the A2AR axis and who might respond to therapeutic blockade of this axis.

## Supplementary Information

Below is the link to the electronic supplementary material.

Supplementary file1 (TIF 1097 KB)

Supplementary file2 (TIF 1075 KB)

Supplementary file3 (TIF 1205 KB)

Supplementary file4 (TIF 1111 KB)

Supplementary file5 (TIF 1110 KB)

Supplementary file6 (TIF 1098 KB)

Supplementary file7 (TIF 1217 KB)

Supplementary file8 (TIF 1179 KB)

Supplementary file9 (TIF 1077 KB)

## Abbreviations

A2AR: Adenosine 2A receptor
ADP: Adenosine diphosphate
AMP: Adenosine monophosphate
APC: Antigen-presenting cell
ATP: Adenosine triphosphate
ccRCC: Clear cell renal cell carcinoma
CD: Cluster of differentiation
CD39: Membrane-bound ectonucleoside triphosphate diphosphohydrolase 1
CD73: Ecto-5′-nucleotidase
chRCC: Chromophobe renal cell carcinoma
coRCC: Collecting duct renal cell carcinoma
CTLA-4: Cytotoxic T-lymphocyte-associated antigen-4
DC: Dendritic cells
HIF: Hypoxia-inducible factor
IC: Infiltrating immune cells
ICI: Immune checkpoints inhibitor
MDSC: Myeloid-derived suppressor cell
MLR: Monocyte-to-lymphocyte ratio
NLR: Neutrophil to lymphocyte ratio
non-ccRCC: Non-clear cell renal cell carcinoma
PD-1: Programmed cell death protein 1
PD-L1: Programmed cell death 1 ligand 1
PLR: Platelet-to-lymphocyte ratio
Prcc: Papillary renal cell carcinoma
pT: Pathological Tumor
RCC: Renal cell carcinoma
RECIST: Response evaluation criteria in solid tumors
SII: Systemic immune-inflammation index
SIRI: Systemic inflammatory response index
TAM: Tumor-associated macrophage
TC: Tumor cells
TFE3-RCC: Renal cell carcinoma with Xp11 translocation involving TFE3 gene fusion
TIL: Tumor-infiltrating lymphocyte
TNM: Tumor-lymph node-metastasis classification of malignant Tumors
Tregs: Regulatory T cells; VEGF: vascular endothelial growth factor
