# Supplementary figures and images for: Increased expression of adenosine 2A receptors in metastatic renal cell carcinoma is associated with poorer response to anti-vascular endothelial growth factor agents and anti-PD-1/Anti-CTLA4 antibodies and shorter survival
Source: Cancer Immunol Immunother. 2021 Jan 8;70(7):2009–21. doi: 10.1007/s00262-020-02843-x (PMC8195893; doi:10.1007/s00262-020-02843-x)

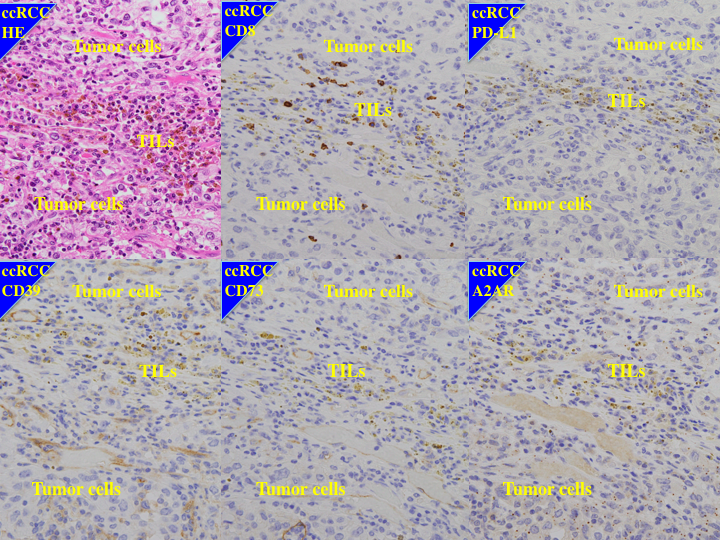

Supplement: Supplementary file 1 — Supplementary file1 (TIF 1097 KB) [file 262_2020_2843_MOESM1_ESM.tif]

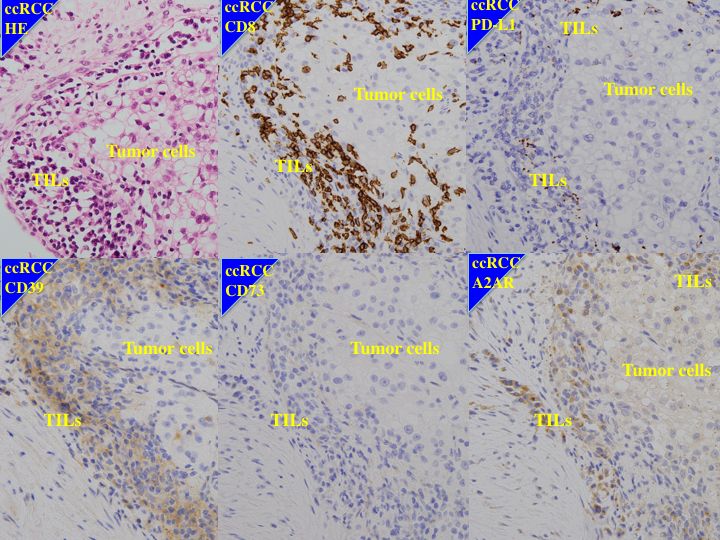

Supplement: Supplementary file 2 — Supplementary file2 (TIF 1075 KB) [file 262_2020_2843_MOESM2_ESM.tif]

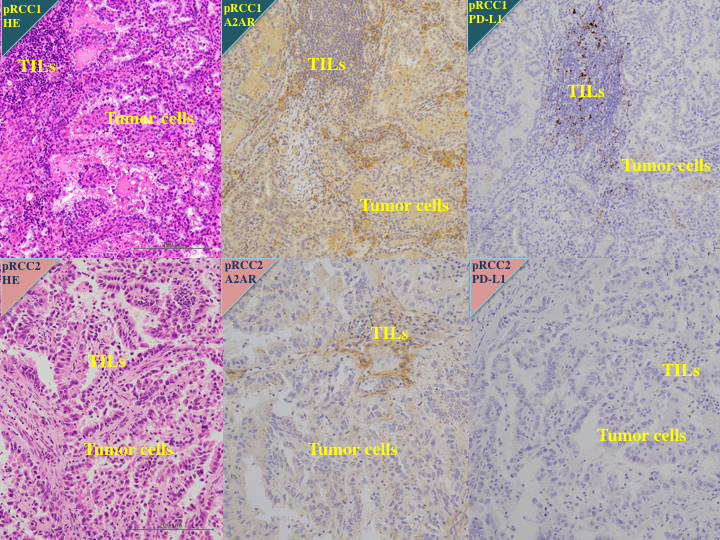

Supplement: Supplementary file 3 — Supplementary file3 (TIF 1205 KB) [file 262_2020_2843_MOESM3_ESM.tif]

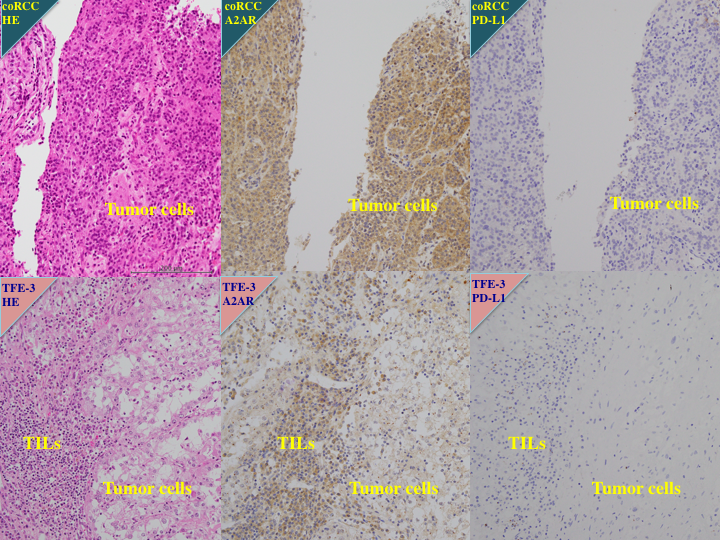

Supplement: Supplementary file 4 — Supplementary file4 (TIF 1111 KB) [file 262_2020_2843_MOESM4_ESM.tif]

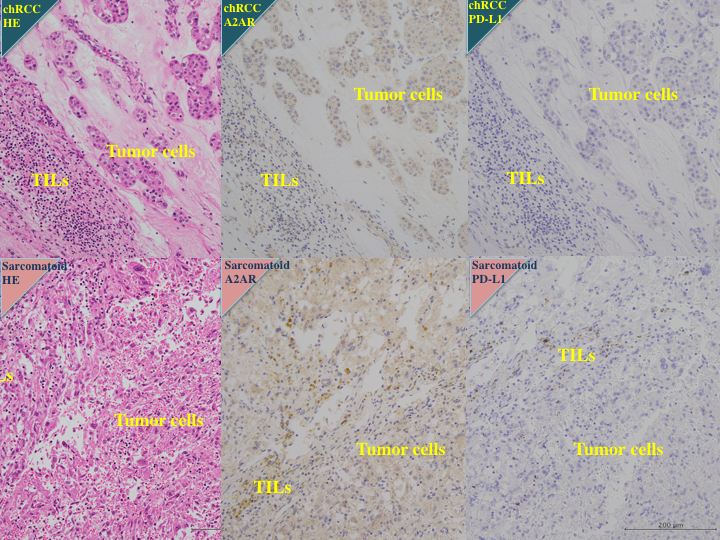

Supplement: Supplementary file 5 — Supplementary file5 (TIF 1110 KB) [file 262_2020_2843_MOESM5_ESM.tif]

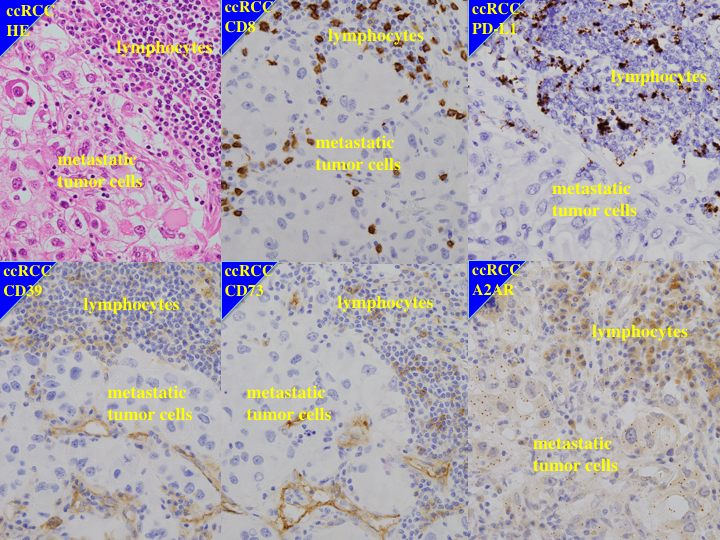

Supplement: Supplementary file 6 — Supplementary file6 (TIF 1098 KB) [file 262_2020_2843_MOESM6_ESM.tif]

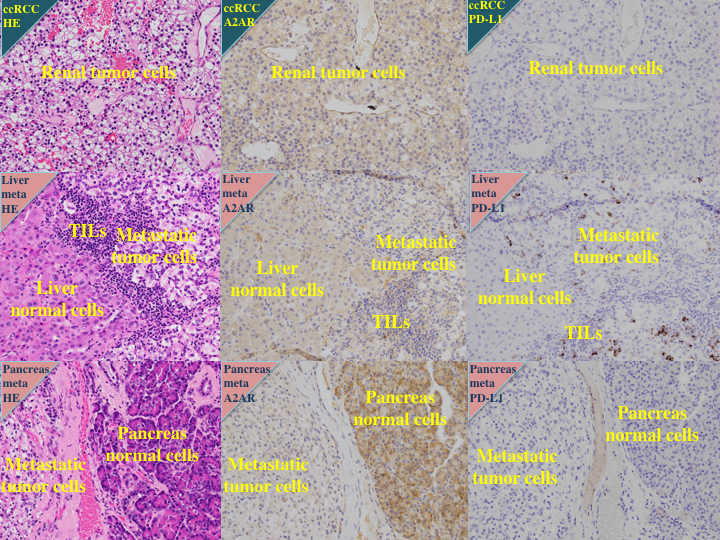

Supplement: Supplementary file 7 — Supplementary file7 (TIF 1217 KB) [file 262_2020_2843_MOESM7_ESM.tif]

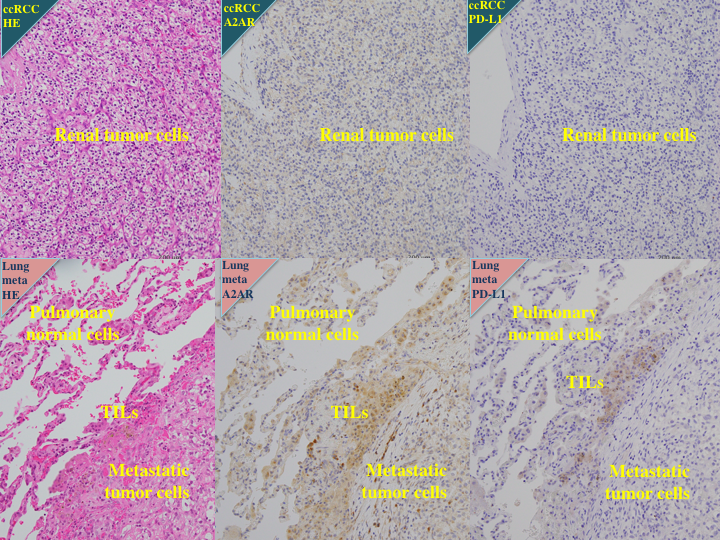

Supplement: Supplementary file 8 — Supplementary file8 (TIF 1179 KB) [file 262_2020_2843_MOESM8_ESM.tif]

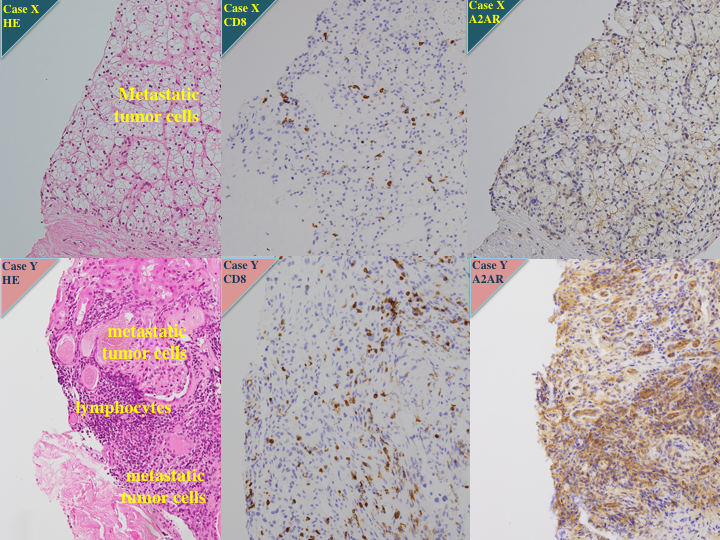

Supplement: Supplementary file 9 — Supplementary file9 (TIF 1077 KB) [file 262_2020_2843_MOESM9_ESM.tif]
